# Source water odor in one reservoir in hot and humid areas of southern China: occurrence, diagnosis and possible mitigation measures

**DOI:** 10.1186/s12302-018-0175-8

**Published:** 2018-11-27

**Authors:** Chao Rong, Dongpo Liu, Yan Li, Kai Yang, Xiaobo Han, Jianwei Yu, Bolun Pan, Jinsong Zhang, Min Yang

**Affiliations:** 1 Shenzhen Water Affairs (Group) Co., Ltd., Water Building, 1019 Shennan Middle Road, Futian District, Shenzhen, Guangdong China; 2 Harbin Institute of Technology Shenzhen Graduate School, HIT Campus of University Town, Nanshan District, Shenzhen, Guangdong China; 3 Civil Engineering, Guangzhou University, Wai Huan Xi Road, Guangzhou Higher Education Mega Center, Guangzhou, Guangdong China; 4 Key Laboratory of Drinking Water Science and Technology, Research Center for Eco-Environmental Sciences, Chinese Academy of Sciences, 18 Shuang Qing Road, Haidian District, Beijing, China

**Keywords:** Drinking water, Earthy–musty odor, *Pseudanabaena* sp., 2-MIB, qPCR

## Abstract

**Background:**

Identifying typical odor-causing compounds is essential for odor problem control in drinking water. In this study, aiming at a major water source reservoir in hot and humid areas in southern China, which encountered seasonable odor problems in recent years, an integrated approach including comprehensive two-dimensional gas chromatography with time-of-flight mass spectrometry (GC × GC–TOFMS), flavor profile analysis (FPA) and quantitative real-time polymerase chain reaction (qPCR) was adopted to investigate the odor occurrence.

**Results:**

The results indicated that earthy–musty odor is blamed to the seasonable odor problems, and it is consistent with the complaints results from consumers. Fifty-four typical odor compounds were investigated in the reservoir and twelve were detected, of which, 2-methylisoborneol (2-MIB) was significantly increased during the odor event. *Pseudanabaena* sp. is the dominant species in the reservoir, which can be further represented by the number of *mic* gene with qPCR method (*R*^2^ = 0.746, *P* < 0.001). Oxygen consumption (COD_Mn_) and dissolved organic carbon (DOC) have great influence on growth of *Pseudanabaena* sp., and the release of 2-MIB from the *Pseudanabaena* sp. cells is affected by temperature and light.

**Conclusion:**

Our findings demonstrated that 2-MIB is the odor-caused substance in the reservoir and *Pseudanabaena* sp. is the main 2-MIB producer, which was confirmed as a benthic filamentous algae. Due to COD_Mn_ and DOC have great influence on *Pseudanabaena* sp. growth, further measures to reduce the CODMn and DOC input should be performed. We also demonstrated that the 2-MIB release is affected by temperature and light. The risk of sudden increase of 2-MIB will be reduced by raising the depth of water in the reservoir. Our study will improve the understanding of T&O problems in this city, as well as in other hot and humid area.

**Electronic supplementary material:**

The online version of this article (10.1186/s12302-018-0175-8) contains supplementary material, which is available to authorized users.

## Background

Taste and odor (T&O) are an important esthetic index to indicate the quality of drinking water and it is a direct hint for users to judge the drinking water whether is safe [1]. Although no researches have shown that the presence of some odorants in the drinking water will cause harm to human health [2–4], the unpleasant smell of drinking water will undoubtedly affect the quality of consumers’ life and the impression of water supply enterprise [5].

Even T&O problem occurrence was widely reported worldwide [4–6], identification of odor causing compounds is still a big challenge. Moreover, due to the low odor threshold concentration (OTC) of the odorants, for instance, 2-MIB (2-methylisoborneol) and geosmin were reported as 10 ng/L and 8 ng/L, respectively [7], and the limited removal efficiency with conventional water treatment process [8, 9], it is difficult for water treatment plant to adopt applicable control measures once encountering T&O episodes in drinking water. For reservoir or lake source water, most T&O occurrence has been linked to some cyanobacterial metabolites such as 2-MIB and geosmin; however, there are still some other possible odorants existence responsible for odor occurrence [10, 11]. Thus, it is one prerequisite to identify possible odorants for further choosing effective control measures.

Gas chromatography and mass spectrometry (GC/MS) have been widely adopted for odorant quantification in water, such as 2-MIB and geosmin [7]. However, due to its limited separation and resolution, it is difficult for odorant qualification. Previous studies have reported that the comprehensive two-dimensional gas chromatography with time-of-flight mass spectrometry (GC × GC–TOFMS) has higher resolution, sensitivity and separation, and it was considered suitable for the analysis of highly complex samples [12]. GC × GC–TOFMS method has more applications in the field of food and chemical industries [13], while having limited use in environment. By combining odor characteristic evaluation, GC × GC–TOFMS has been applied for odorant screening and identification in complex aquatic environment [14].

Because the algae growth, synthesis and release of odorants in algae cells are affected by environmental factors such as water temperature, precipitation, light, and nutrients [15–17], identifying the odorant-producing algae and finding the key environmental affecting factors can reduce the risk of T&O outbreaks from the source. Su et al. [18] indicated that the risk for T&O could be reduced by increasing the water level in Miyun reservoir (2.91% reduced for each meter increase). However, it should be noted that this process is difficult since there are many species in the reservoir, and not all phytoplankton produce these compounds, such as 2-MIB and geosmin; < 50 of the more than 2000 species classified to date have been directly confirmed as producers [19]. In recent years, the genes involved in the synthesis of odorants have studied a lot, and detection of odorants-producing algae with qPCR method has been widely used [20–22]. Wang et al. have revealed genes involved in cyanobacteria and first studied the correlation between the gene (*mic*) and 2-MIB concentration (10–60 fg 2-MIB per *mic* copy) [22]. This provides a new way to identify the odorants-produced algae and must be more accurate than previous method.

Shiyan reservoir (SY) is one major water source of Shenzhen city, which is a representative water resource in hot and humid regions of China. In recent years, the reservoir has encountered seasonable T&O problems, especially in April to July, which is one typical high temperature and rainy season. Even one typical odorant of 2-MIB was ever detected sporadically, the key odorants, occurrence procedure and cause were not clear. In this study, by comparing to one nearby reservoir with no odor occurrence, the odor occurrence, possible odorants, odor-producing algae and possible control measures for reducing odor occurrence risk in SY reservoir were systematically investigated. The results of this study will be helpful for further improvement of the drinking water quality and water management suffering from odor problems.

## Methods

### Field sites and sampling

Shiyan reservoir (SY) was located at the northwest part of Shenzhen City, with a catchment area of 44 km^2^ and a corresponding storage capacity of 16.9 million m^3^. Every April to July, odor problem would occur about 4 months. For comparison, Shenzhen reservoir (SZ) was selected as reference, where no odor occurrence was recorded, with a catchment area of 60.5 km^2^ and a storage capacity of 45.77 million m^3^.

Samples were taken from the intake (0.5 m below the surface) of two reservoirs from October 2016 to May 2018, and total of 66 samples were collected. Samples were collected weekly in the spring and summer (April to September), and approximately monthly at other times of the period throughout 2016 and 2018. The samples from October 28, 2016, May 8, 2017 (odor event) and September 26, 2017 were used for odorant identification analysis.

**Fig. 1:**
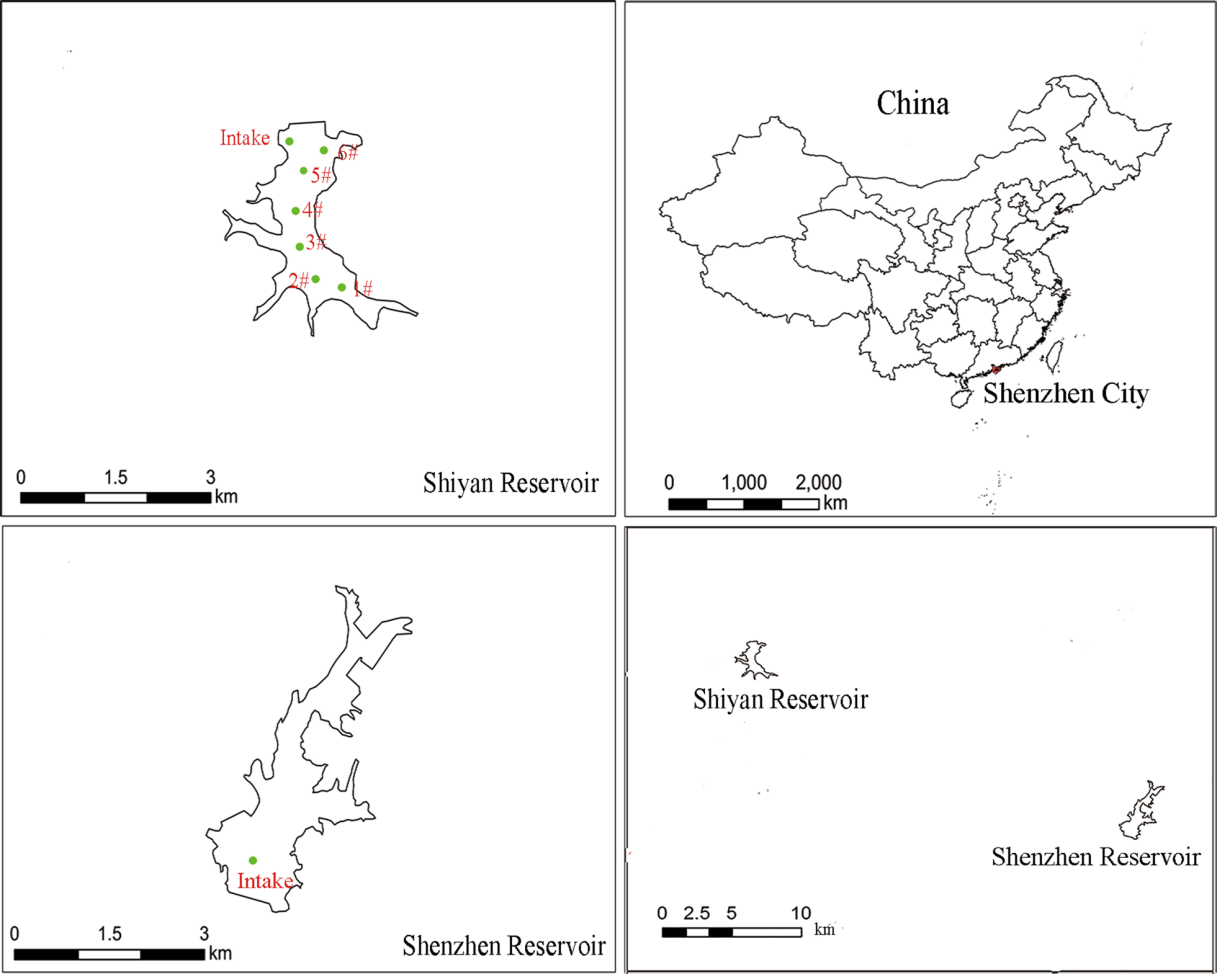
The sampling sites in Shiyan reservoir and Shenzhen reservoir

During the April to July, 2018, additional sampling sites (1–6#) were added in SY. Sample sites 1–2# were close to the water inlet of the reservoir and the water depth is only about 3 meters. For other sample sites, the water depth is > 10 m. The water samples from the surface layer (0.5 m below the surface) and bottom layer (0.5 m above the sediment) were analyzed. The sampling sites of the two reservoirs are shown in Fig. 1.

While sampling, 1 L of water is added to MgCO_3_ turbid solution (prevention of pigment decomposition) for chlorophyll-a analysis, 1 L of water is added to 5% Lugol’s iodine for algae qualification and cell counting, 1 L of water (no head space) is added HgCl_2_ solution for the odorant determination, and another 5 L of water was collected for qPCR analysis and other water parameters. Values of water temperature, pH, dissolved oxygen and turbidity were obtained in situ. Water samples for odorants and other physio-chemical parameters analysis were transported to the laboratory immediately in a portable refrigerator (around 4 °C). A total of 11 typical physio-chemical parameters were analyzed and analysis methods are shown in Table 1.

**Table 1:** Physio-chemical indicators and analysis methods

Indicator	Analysis methods
Water temperature	Thermo ORION 3 STAR
pH	Thermo ORION 3 STAR
DO	HACH HQ 30d
Chroma	XINRUI-SD9012AB
Turbidity	HACH-2100AN
UV_254_	VARIAN-CARY50
DOC	GE 5310CTOC
COD_Mn_	Acidic potassium permanganate titration
TN	Alkaline potassium persulfate
TP	Molybdate spectrophotometric method
Chlorophyll-a	Thermal ethanol extraction spectrophotometric method

### Odor evaluation

Flavor profile analysis (FPA) was employed for odor evaluation. A detailed description of the training and applications for the FPA method can be found in the standard methods for water and wastewater [23]. The panels were made up of at least four panelists for each test. Seven-point scales of 1–12 were used to describe the intensity of the samples [(1) odor threshold, (2)–(4) weak odor intensity, (6)–(8) moderate odor intensity, and (10)–(12) strong odor intensity]. Odor standards with different intensities were used to remind the panel of the odor descriptors and intensities with each batch of samples.

### OAV determination

Odor perception in water depends not only on the concentration of odor substances, but also on the odor threshold concentration (OTC). Odor activity value (OAV), as a method of odor characterization, is defined as the ratio of odorant concentration to its threshold, which involves in the influence of odor concentration and odor threshold concentration [24]. When OAV > 1, it means that the concentration of the compound is greater than its OTC, and has a greater contribution to the odor intensity of water. The higher the OAV is the more contribution to the odor profile [25]. While OAV < 1, it means that the concentration of the substance is lower than its OTC, and it contributes less to the odor intensity of water.

### GC × GC–TOFMS analysis

The water sample was pretreated with liquid–liquid extraction. The specific steps are as follows: After the water sample was filtered through a 1.2 μm glass fiber membrane (GF/C), 500 mL water samples were extracted using dichloromethane (HPLC grade) twice (50 mL and 30 mL for the first and second extraction, respectively) and then dehydration was carried out with Na_2_SO_4_; the samples were concentrated to a final volume of 100 μL, followed by rotary evaporation and blowing off under a gentle nitrogen stream. The pressure of the rotary evaporation chamber was 920 mbar, while the temperatures of the water bath and cooling cycling water were 28 °C and 10 °C, respectively. The overall preconcentration factor was 5000 before the analysis sample in − 20 °C stored in fridge.

Odor substances were analyzed by GC × GC–TOFMS. In the first dimension, a low polarity capillary column was used (Rxi-5silv 30 m × 0.25 mm × 0.25 µm), while in the second dimension column was a polar one (RXI-17 1.79 m × 0.1 mm × 0.1 µm). Ultrapure helium (He ≧ 99.999%) was used as the carrier gas at the constant flow of 1 mL/min. 1 mL extracts were introduced using a programmed temperature vaporizing injector at 50 mL/s in a splitless mode with the inlet temperature of 250 °C. The temperature program of the first column (main GC oven) was optimized as follows: 40 °C (0.2 min) → 280 °C (at 5 °C/min) → 280 °C (5 min). The temperature of the second oven was programmed from 45 °C (0.2 min) to 285 °C at a rate of 5 °C/min with a final hold time of 5 min. The transfer line linking the secondary oven with the mass spectrometer was maintained at 250 °C. The electron energy was 70 eV, and the detector voltage was set at 1575 eV. The data acquisition rate was 100 spectra per s, covering a mass range of 50–500 *m*/z. The temperature of the ion source was set at 250 °C [14] (Table 2).

**Table 2:** Information of the 54 odor compounds analyzed by GC × GC–TOFMS

No.	Compounds	Odor description	OTC (mg/L)	CAS
1	Hexanal	Herbal flavor, almond	4.5	66-25-1
2	Heptanal	Fishy	3.0	111-71-7
3	Benzaldehyde	Herbal flavor	4.5	100-52-7
4	2,4-Heptadienal	Fishy/oily	5.0	4313-03-5
5	2-Octenal	Irritant	n.a.	2548-87-0
6	Nonanal	Fruity, fragrance	n.a.	124-19-6
7	2,6-Nonadienal	Herbal flavor/cucumber	0.08	17587-33-6
8	Decanal	Orange flavor	n.a.	112-31-2
9	2,4-Decadienal	Oily	0.029	2363-88-4
10	2,6,6-Trimethyl-1-cyclohexene-1-carboxaldehyde	Sweet, fragrance	n.a.	432-25-7
11	Ethylbenzene	Plastic, oily, chemical	150.0	100-41-4
12	p-Xylene	Chemical	n.a.	106-42-3
13	1,4-Dichloro-benzene	Almond, sweet	4.5	106-46-7
14	1,3,5-Trichloro-2-methoxy-benzene	Musty	0.002	108-70-3
15	Bis(2-chloroisopropyl) ether	Medicinal odor	0.017	39638-32-9
16	Butanoic acid, propyl ester	Alcohol	n.a.	105-66-8
17	Indole	Stinky	0.1	120-72-9
18	3-Methyl-indole	Stinky	1.0	83-34-1
19	Ionone	Fragrance	0.007	8013-90-9
20	Tetramethyl pyrazine	Sour, fragrance	2.6	1124-11-4
21	Pyrazine	Fragrance	2.7	290-37-9
22	2-Methoxy-3-(2-methylethyl)-pyrazine/IPMP	Musty	0.0002	25773-40-4
23	2-Methoxy-3-(2-methylpropyl)-pyrazine/IBMP	Musty	0.001	24683-00-9
24	Pyridine	Amine, stinky	1.1	110-86-1
25	2-Methyl-phenol	Medicinal odor	14.7	95-48-7
26	4-Bromo-phenol	Medicinal odor	n.a.	106-41-2
27	3-Methyl-phenol	Medicinal odor	12.8	108-39-4
28	2-Nitro-phenol	Medicinal odor	11.0	88-75-5
29	2,6-Dimethyl-phenol	Medicinal odor, musty	11.0	576-26-1
30	2-Chloro-phenol	Chemical, musty, floral	0.088	95-57-8
31	Dimethyl sulfide	Rotten cabbage	1.0	75-18-3
32	Diethyl sulfide	Swampy, septic	n.a.	352-93-2
33	Dimethyl disulfide	Swampy, septic	0.03	624-92-0
34	Diisopropyl sulfide	Swampy, septic	n.a.	625-80-9
35	Propyl sulfide	Swampy, septic	0.0019	111-47-7
36	Diethyl disulfide	Swampy, septic	0.02	110-81-6
37	Dimethyl trisulfide	Swampy, septic	0.01	3658-80-8
38	Butyl sulfide	Swampy, septic	0.00189	544-40-1
39	Dipropyl disulfide	Swampy, septic	n.a.	629-19-6
40	Amyl sulfide	Swampy, septic	0.0011	872-10-6
41	Dibutyl disulfide	Swampy, septic	n.a.	629-45-8
42	Dipentyl disulfide	Swampy, septic	n.a.	112-51-6
43	Benzyl disulfide	Foul smell	n.a.	150-60-7
44	1-Pentanethiol	Rancid, stinky	n.a.	110-66-7
45	1-Heptanethiol	Rancid, stinky	n.a.	1639-09-4
46	1-Octanethiol	Rancid, stinky	n.a.	111-88-6
47	1-Nonanethiol	Rancid, stinky	n.a.	1455-21-6
48	Thiomorpholine	Fishy, stinky	n.a.	123-90-0
49	Thiazole	Foul smell	n.a.	288-47-1
50	Pentachlorothioanisole	Medicinal	n.a.	1825-19-0
51	Indane	Musk, fragrance	n.a.	496-11-7
52	Eucalyptol	Peppermint	n.a.	470-82-6
53	2-Methylisoborneol	Musty	0.01	2371-42-8
54	Geosmin	Earthy	0.004	19700-21-1

### Algal enumeration

The water sample was added 5% Lugol’s iodine and static settlement by 48 h, then pre-concentrated 100× and kept in dark until cell counting [26]. The algal cell density was determined by the Utermöhl technique using a Sedgewick-Rafter counting chamber under a microscope (Nikon Eclipse 50i) with phase contrast and bright field illumination [27]. A magnification of 160× was used to enumerate the cells. Triplicates of 1 mL concentrated samples were collected separately and counted. The number of filamentous algal cells was calculated by dividing the measured filamentous length by the mean cell length; the number of cells in colony species such as *Microcystis* sp. was estimated based on colony volume and mean cell density.

### Real-time PCR

The genomic DNA was extracted from *Pseudanabaena* sp. (FACHB1277) which is 2-MIB producer. Field samples were filtered through 0.22 μm polycarbonate filter (GTTP type, Millipore, USA) and cultured algae was centrifuged for 10 min under 4000×*g* before genomic DNA extraction. The Fast DNA^®^SPIN KIT for Soil (MP Biomedicals, USA) kit was used and operated according to the manufacturer’s instructions.

The primer pair MIB-R(f/r)(MIB-Rf 5′-CGACAGCTTCTACAYCYCCATGAC-3′, MIB-Rr 5′-CGCCGCAATCTGTAGCACCAT-3′) was used to amplify the *mic* fragments [22]. The PCR instrument used was C1000™ Thermal Cycler (Bio-Rad, USA). PCR amplification was carried out in 1 × DynaZyme II buffer (Thermo Scientific, USA) with 0.2 mM of each primer, 200 mM of dNTPs, 0.4 U of DynaZyme II, and 20–50 ng of genomic DNA as template. The PCR protocol was 94 °C for 3 min, 35 cycles of 94 °C for 30 s, 59 °C for 30 s, 72 °C for 60 s, and 72 °C for 5 min. Then, the amplification product is subjected to a purification operation with SanPrep KIT (Sangon Biotech, China) and performed according to the manufacturer’s instructions. The size of the PCR products was checked by gel electrophoresis using a 1.5% agarose gel and a l-*Hin*dIII/Фx-*Hae*II DNA marker (Thermo Scientific, USA). The concentration and purity of the product were measured with a NanoDrop ND-1000 spectrophotometer (Thermo Scientific, USA) and then used in qPCR standard curve built.

The 20 μL of SYBR Green qPCR systems contained 1 μL of genomic DNA of cultured strains or environmental samples, 0.6 μM of final concentration of MIB-R(f/r), 10 μL of hot start reaction mix containing SYBR Green I (Gen Star, China), and 8 μL of ddH_2_O. Amplification was performed in a 7500 real-time system (ABI, USA) with the following program: an initial preheating step of 10 min at 95 °C, followed by 35 cycles, with one cycle consisting of 15 s at 95 °C and 30 s at 59 °C. To determine melting temperatures for the amplification products, the temperature was raised after amplification from 70 to 95 °C, and fluorescence was continuously detected.

## Results and discussion

### Identification of odor-caused substances

During the odor event on May 8, 2017, a total of 43 complaints related to T&O problems in drinking water were received from consumers. However, the overwhelming majority (62.79%, 27) of people could not to precise describe the odor type. This may be due to a lack of T&O training and live in different environments [28–30]. To identify the odor type exactly, raw water samples from two reservoirs were evaluated by FPA method and the results were compared with the other two reports without odor problem (October 28, 2016 and September 26, 2017). The results are shown in Table 3. There are weak (3–4) intensity earthy–musty and swampy/septic odor in SZ, and no significant changes during the period of odor event on May 8, 2017. The major odor types of SY are earthy–musty and grassy, and the intensity was moderate in the odor event. Compared with usual conditions, the odor intensity of SY increased obviously (earthy–musty intensity from 5 to 7, grassy intensity from 6 to 7). The main odor type in Shenzhen is earthy–musty, which is different from the previous studies on the odor and taste of fresh water source in other areas of China, for example, the septic and marshy odor in Wuxi city, and fishy odor in a north China reservoir [4, 31].

**Table 3:** FPA evaluation results of two reservoirs

Reservoir	28/10/2016	08/05/2017	26/09/2017
SZ	Earthy–musty (3)^a^ Swampy/septic (4)	Earthy–musty (3) Swampy/septic (3)	Earthy–musty (3) Swampy/septic (4)
SY	Earthy–musty (5) Grassy (6)	Earthy–musty (7) Grassy (7)	Earthy–musty (4) Grassy (3)

^a^Odor type (intensity)

To determine the main odor-causing substances in the water source, 54 odorants known to cause common T&O problems in water sources were analyzed by GC × GC–TOFMS, and the results are shown in Table 4. In the two reservoirs, a variety of odor compounds were detected, 10 compounds in SZ and 12 compounds in SY. The main odor substances include earthy–musty substances (2-MIB and geosmin), swampy/septic substances (dimethyl disulfide). Before and after the odor event, there was no change in the type of odorant, but the concentration of some odorants changed. For earthy–musty substances, only geosmin was detected in the SZ. While both 2-MIB and geosmin were detected in SY, and the concentration of 2-MIB was much higher than that of geosmin (2-MIB and geosmin mean values are 36.95 ng/L and 5.54 ng/L in SY, respectively). During the odor event (May 8, 2017), the 2-MIB concentration in SY is 52.87 ng/L, which is much higher than the other times (35.85 ng/L in October 28, 2016 and 22.13 ng/L in September 26, 2017), but the geosmin concentration was unchanged and remained low.

**Table 4:** GC × GC–TOFMS analysis results of two reservoirs

No	Compounds	28/10/2016		08/05/2017		26/09/2017	
		SZ	SY	SZ	SY	SZ	SY
1	Hexanal	n.d.	n.d.	n.d.	n.d.	n.d.	n.d.
2	Heptanal	n.d.	n.d.	n.d.	n.d.	n.d.	n.d.
3	Benzaldehyde	n.d.	n.d.	n.d.	n.d.	n.d.	n.d.
4	2,4-Heptadienal	n.d.	n.d.	n.d.	n.d.	n.d.	n.d.
5	2-Octenal	n.d.	n.d.	n.d.	n.d.	n.d.	n.d.
6	Nonanal	n.d.	n.d.	n.d.	n.d.	n.d.	n.d.
7	2,6-Nonadienal	n.d.	n.d.	n.d.	n.d.	n.d.	n.d.
8	Decanal	n.d.	n.d.	n.d.	n.d.	n.d.	n.d.
9	2,4-Decadienal	n.d.	n.d.	n.d.	n.d.	n.d.	n.d.
10	2,6,6-Trimethyl-1-cyclohexene-1-carboxaldehyde	5.75	24.47	9.11	26.42	5.86	23.32
11	Ethylbenzene	n.d.	n.d.	n.d.	n.d.	n.d.	n.d.
12	p-Xylene	n.d.	6.15	n.d.	5.95	n.d.	3.21
13	1,4-Dichloro-benzene	1.11	n.d.	0.31	n.d.	1.32	n.d.
14	1,3,5-Trichloro-2-methoxy-benzene	n.d.	n.d.	n.d.	n.d.	n.d.	n.d.
15	Bis(2-chloroisopropyl) ether	n.d.	n.d.	n.d.	n.d.	n.d.	n.d.
16	Butanoic acid, propyl ester	n.d.	n.d.	n.d.	n.d.	n.d.	n.d.
17	Indole	n.d.	n.d.	n.d.	n.d.	n.d.	n.d.
18	3-Methyl-indole	n.d.	n.d.	n.d.	n.d.	n.d.	n.d.
19	Ionone	n.d.	n.d.	n.d.	n.d.	n.d.	n.d.
20	Tetramethyl pyrazine	1.33	1.18	1.11	1.23	1.07	1.54
21	Pyrazine	n.d.	n.d.	n.d.	n.d.	n.d.	n.d.
22	2-Methoxy-3-(2-methylethyl)-pyrazine/IPMP	n.d.	n.d.	n.d.	n.d.	n.d.	n.d.
23	2-Methoxy-3-(2-methylpropyl)-pyrazine/IBMP	n.d.	n.d.	n.d.	n.d.	n.d.	n.d.
24	Pyridine	33.06	22.95	45.72	24.63	37.09	21.89
25	2-Methyl-phenol	2.86	9.17	2.46	8.93	2.03	9.24
26	4-Bromo-phenol	n.d.	n.d.	n.d.	n.d.	n.d.	n.d.
27	3-Methyl-phenol	5.52	4.20	4.97	5.32	4.03	3.79
28	2-Nitro-phenol	n.d.	n.d.	n.d.	n.d.	n.d.	n.d.
29	2,6-Dimethyl-phenol	n.d.	n.d.	n.d.	n.d.	n.d.	n.d.
30	2-Chloro-phenol	n.d.	n.d.	n.d.	n.d.	n.d.	n.d.
31	Dimethyl sulfide	n.d.	n.d.	n.d.	n.d.	n.d.	n.d.
32	Diethyl sulfide	n.d.	n.d.	n.d.	n.d.	n.d.	n.d.
33	Dimethyl disulfide	8.48	0.34	3.21	n.d.	6.79	0.56
34	Diisopropyl sulfide	n.d.	n.d.	n.d.	n.d.	n.d.	n.d.
35	Propyl sulfide	n.d.	n.d.	n.d.	n.d.	n.d.	n.d.
36	Diethyl disulfide	n.d.	n.d.	n.d.	n.d.	n.d.	n.d.
37	Dimethyl trisulfide	n.d.	n.d.	n.d.	n.d.	n.d.	n.d.
38	Butyl sulfide	n.d.	n.d.	n.d.	n.d.	n.d.	n.d.
39	Dipropyl disulfide	n.d.	n.d.	n.d.	n.d.	n.d.	n.d.
40	Amyl sulfide	n.d.	n.d.	n.d.	n.d.	n.d.	n.d.
41	Dibutyl disulfide	n.d.	n.d.	n.d.	n.d.	n.d.	n.d.
42	Dipentyl disulfide	n.d.	n.d.	n.d.	n.d.	n.d.	n.d.
43	Benzyl disulfide	n.d.	n.d.	n.d.	n.d.	n.d.	n.d.
44	1-Pentanethiol	5.58	6.95	6.73	7.32	5.65	7.57
45	1-Heptanethiol	n.d.	n.d.	n.d.	n.d.	n.d.	n.d.
46	1-Octanethiol	n.d.	n.d.	n.d.	n.d.	n.d.	n.d.
47	1-Nonanethiol	n.d.	n.d.	n.d.	n.d.	n.d.	n.d.
48	Thiomorpholine	n.d.	n.d.	n.d.	n.d.	n.d.	n.d.
49	Thiazole	8.25	20.9	8.75	21.35	9.02	22.09
50	Pentachlorothioanisole	n.d.	n.d.	n.d.	n.d.	n.d.	n.d.
51	Indane	n.d.	n.d.	n.d.	n.d.	n.d.	n.d.
52	Eucalyptol	n.d.	1.82	n.d.	2.01	n.d.	1.53
53	2-Methylisoborneol	n.d.	35.85	n.d.	52.87	n.d.	22.13
54	Geosmin	2.04	1.65	4.21	7.98	1.21	6.99

**Fig. 2:**
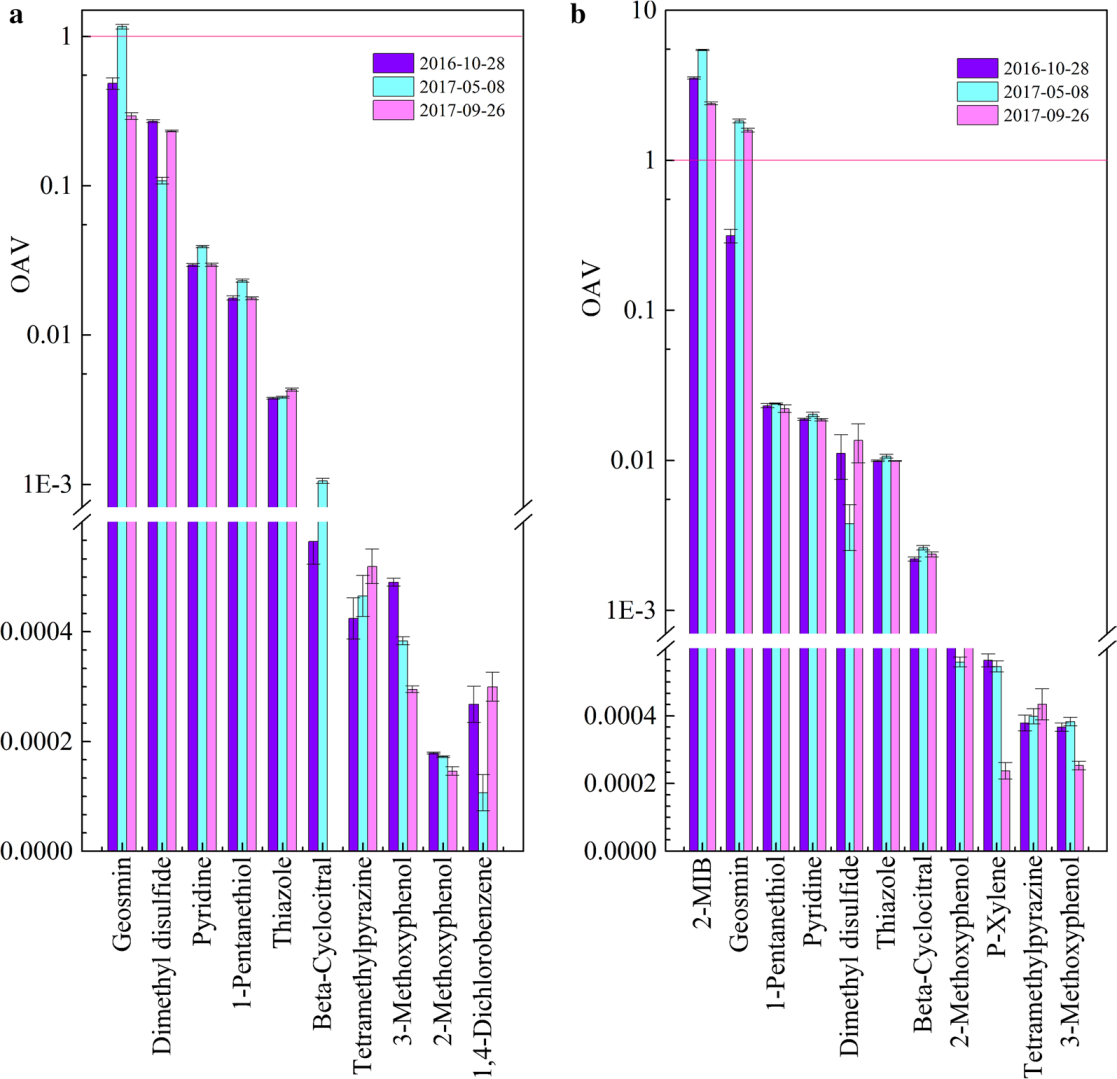
OAV ranking of the detected odorants in two reservoirs: **a** Shenzhen reservoir, **b** Shiyan reservoir

To characterize the contribution of each compound to the odor intensity of water in a more intuitive way, we calculated the odor activity value (OAV) of each substance which was detected in the two reservoirs and the results are shown in Fig. 2. The OAVs of detected substances in SZ were almost less than 1 (only the OAV of geosmin is 1.16 in May 8, 2017), indicating that each substance contributed less to the overall odor intensity in water, which was consistent with the FPA results. Compared with SZ, a much higher OAV of 2-MIB occurred in SY, which increased significantly to 5.29 during the odor event from 3.59 and 2.21 in other two periods. This implies that 2-MIB was responsible for the earthy musty problem occurrence.

The FPA result (Table 3) also shows that there is moderate odor intensity grassy smell in SY, while the GC × GC–TOFMS results show that the grassy compounds were undetected in the reservoir. This may be the substances out of the list that cause the grassy odor [32–34]. There were no complaints related to grassy odor from consumers. This may be due to the fact that the weak grassy odor is easily masked by disinfectants in the finished water [35, 36]. In addition, several other odor compounds such as 2,6,6-trimethyl-1-cyclohexene-1-carboxaldehyde, pyridine, 2-methyl-phenol and thiazole were detected in two reservoirs, but the OAVs of these substances are below < 1; it means that these compounds have limited contribution to T&O in the reservoirs.

### Identification of 2-MIB-producing algae

To identify the 2-MIB-producing algae in Shenzhen area, the phytoplankton changing of two reservoirs was detected by microscope. Five phyla, including Cyanophyta, Chlorophyta, Bacillariophyta, Euglenophyta and Cryptophyta, were detected in the two reservoirs. The total phytoplankton cell abundance in SY was much higher than SZ during the monitoring period (Additional file 1: Figure S1). The peak value of phytoplankton cells in SY is 272.07 × 10^6^ cells/L, while the highest value in SZ is only 5.35 × 10^6^ cells/L. The relative abundance of phytoplankton in SZ and SY is also different (Additional file 1: Figure S2), Cyanophyta was the dominant phylum in SY in most of the year, while the dominant phytoplankton in SZ varies significantly. Cyanophyta, Chlorophyta and Bacillariophyta form the dominant groups in different months. The main algae microscopy images of the reservoirs are shown in Additional file 1: Figure S3.

**Fig. 3:**
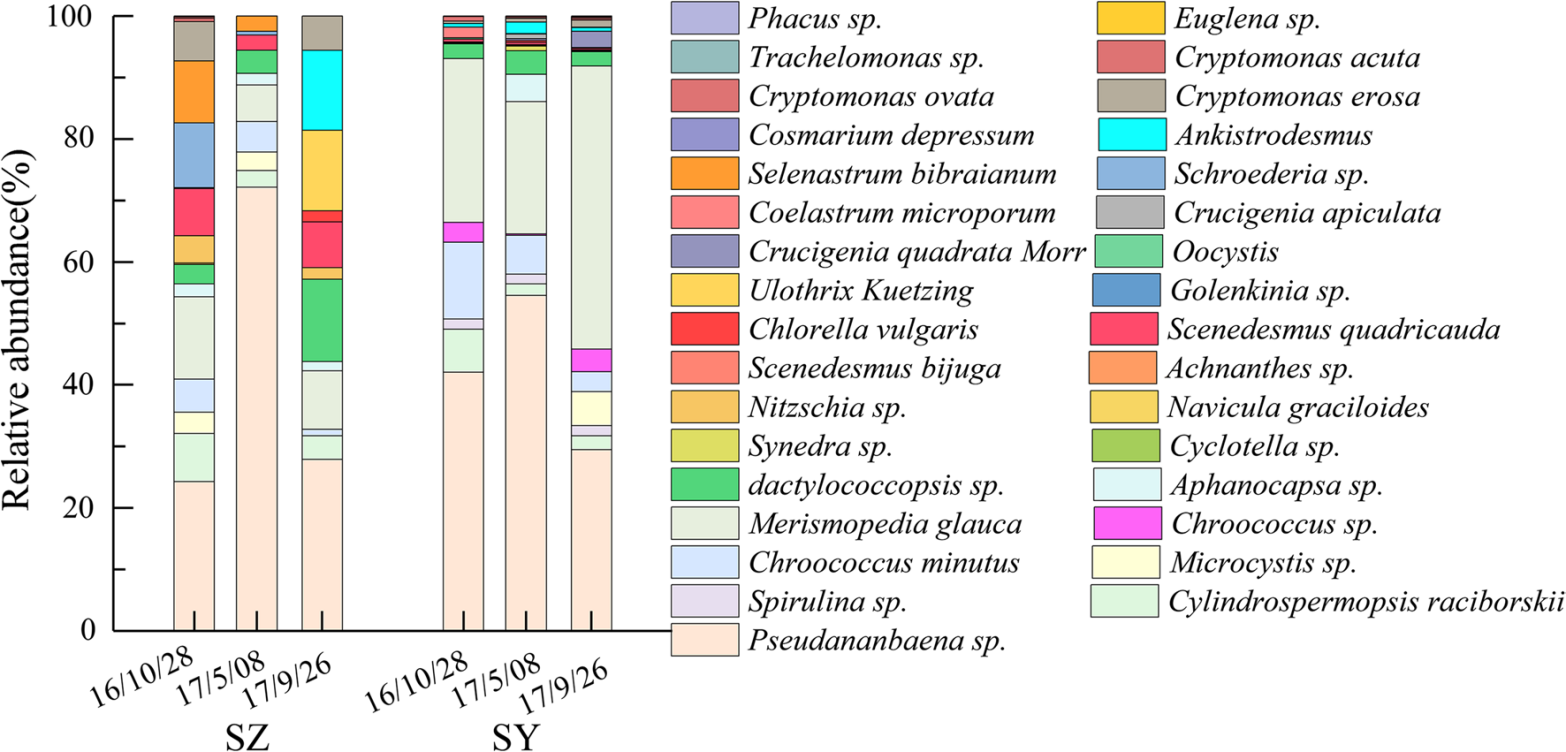
The variation of phytoplankton in SZ and SY before, after, and during the odor event by genus

The phytoplankton data from the two reservoirs on May 8, 2017, October 28, 2016 and September 26, 2017 are shown in Fig. 3. When the odor event occurred, the total amount of algae in SY was as high as 100 million, which was significantly higher than the other two times. Although the algae counts in SZ also increased, they remained much lower than the algae level in SY. During the odor event, the relative abundance of some cyanobacteria species, especially *Pseudanabaena* sp., increased significantly. In previous studies, *Pseudanabaena* sp. has been identified as one of the 2-MIB producers in the aquatic ecosystem, and it is suggested that as little as 27–64 filaments per milliliter could impart an earthy–musty odor to the water with each filament containing 30 cells [37, 38]. Therefore, it can be inferred that 2-MIB in the reservoir was mainly produced by the *Pseudanabaena* sp.

The relationship between the total algae biovolume (represented by chlorophyll-a) and 2-MIB was analyzed, and the correlation was weak (0.349, *P* < 0.05) (Table 5). This indicates that the total algae counts cannot reflect the change of 2-MIB in the water, while correlating *Pseudanabaena* sp. with 2-MIB for further analysis (Additional file 1: Figure S4). The correlation coefficient was significantly higher than before (0.766, *P* < 0.01) (Table 6). This also indicates that *Pseudanabaena* sp. is probably the source of 2-MIB.

**Table 5:** Correlation analysis of chlorophyll-a and 2-MIB of SY

		Chlorophyll-a	2-MIB
Chlorophyll-a	Pearson correlation	1	0.349^a^
	Sig. (2-tailed)		0.046
	N	33	33
2-MIB	Pearson correlation	0.349^a^	1
	Sig. (2-tailed)	0.046	
	N	33	33

^a^Correlation is significant at the 0.05 level (2-tailed)

**Table 6:** Correlation analysis of chlorophyll-a and 2-MIB of SY after data selected

		Chlorophyll-a	2-MIB
Chlorophyll-a	Pearson correlation	1	0.766^a^
	Sig. (2-tailed)		0.000
	N	19	19
2-MIB	Pearson correlation	0.766^a^	1
	Sig. (2-tailed)	0.000	
	N	19	19

^a^Correlation is significant at the 0.01 level (2-tailed)

**Fig. 4:**
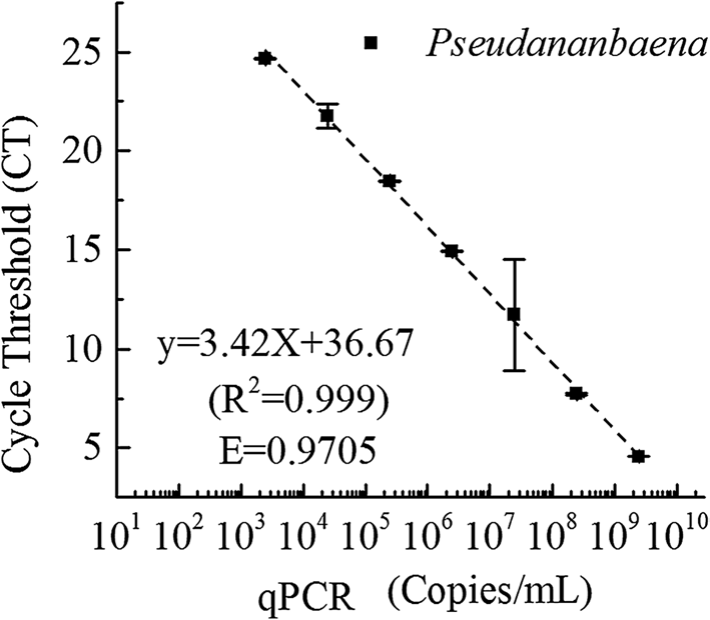
Standard curve used for the qPCR assay

The qPCR method was used to verify whether or not *Pseudanabaena* sp. is the 2-MIB producer in the reservoir. The genomic DNA was extracted from laboratory-cultured *Pseudanabaena* sp. (FACHB-1277) and used as genomic DNA after amplification and purification. The gel electrophoresis result of amplification products before and after purification operation is shown in Additional file 1: Figure S5. The concentration of product after purification was determined to be 10.9 ng/μL, and the gene copy number *N* was determined to be 4.92 × 10^10^ copies/μL. Figure 4 shows that PCR standards were successfully amplified for primer MIB-R(f/r), with the range of 2.46 × 10^3^–2.46 × 10^9^ copies/μL. The standard curve shown in the figure is linear with very high correlation (*R*^*2*^ = 0.999) and the high efficiency (*E *= 0.9705). This means that the amplicon quantity nearly doubled every cycle and the amplification efficiency was reasonable for quantification.

**Fig. 5:**
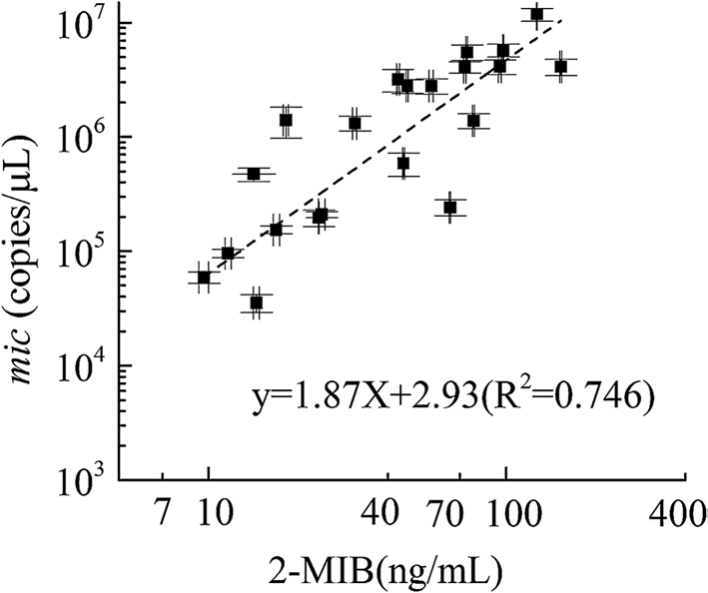
Relationship between 2-MIB concentrations and *Pseudanabaena* sp. 2-MIB synthase gene (mic)

The relationship between the number of *mic* and 2-MIB concentration in the filed simples is shown in Fig. 5. The results indicated that the number of *mic* genes was closely correlated with 2-MIB concentration by linear regression (*R*^*2*^ = 0.746, *P * < 0.001), and show that one *mic* represent about 10 fg 2-MIB, which is similar to previous study [22].

The quantitative detection of *mic* gene can reflect the formation potential of 2-MIB in DNA level and may be served as an early warning method for 2-MIB event in the reservoir. Because previous studies found that total cyanobacteria biovolume is not a consistent predictor of geosmin, and correlation between a particular taxon and particle-bound geosmin cannot be expected to occur in any instance in natural waters. This study also found a similar phenomenon in 2-MIB (Table 5). Because *Pseudanabaena* sp. is the main source of 2-MIB, the quantity of *Pseudanabaena* sp. can reflect the ability of 2-MIB production. However, algae enumeration through microscopes is time consuming and requires strong technical/professional skills. Although this qPCR method does not explain the production of 2-MIB gene expression in RNA level, compared with the observation of *Pseudanabaena* sp. by microscope, it is more accurate and has higher sensitivity.

### Possible measures to reduce 2-MIB occurrence risk

The production of 2-MIB and geosmin by cyanobacteria can be influenced by many factors, such as temperature, light intensity, and nutritional factors [17, 39, 40]. As the *Pseudanabaena* sp. is the source of 2-MIB in the reservoir, it is essential to prevent the growth of *Pseudanabaena* sp. in order to reduce the risk of 2-MIB odor event occurrence in raw water sources. Therefore, it is imperative to assess the favorite habitat of *Pseudanabaena* sp. in SY. Long time monitoring of SZ and SY was conducted from October 2016 to May 2018. The temporal distribution of physio-chemical parameters is shown in Table 7.

**Table 7:** Major physio-chemical variables in two reservoirs during study period

Season	Area	Water Temp. (^o^C)	Chroma	Turbidity (NTU)	PH	DO (mg/L)	UV_254_	DOC (mg/L)	COD_Mn_ (mg/L)	TN (mg/L)	TP (mg/L)	Chl-a (μg/L)
Spring	SZ	23.00 ± 2.63	15.86 ± 4.75	3.97 ± 1.58	7.53 ± 0.34	9.72 ± 0.81	0.03 ± 0.01	2.52 ± 0.95	2.38 ± 0.39	4.10 ± 1.60	0.09 ± 0.03	14.72 ± 6.64
	SY	24.64 ± 2.96	21.93 ± 5.93	4.26 ± 0.79	8.50 ± 0.82	10.12 ± 1.9	0.03 ± 0.01	3.39 ± 2.14	3.88 ± 1.02	3.92 ± 1.47	0.08 ± 0.04	40.65 ± 15.67
Summer	SZ	28.73 ± 0.58	16.05 ± 2.12	5.71 ± 1.99	7.77 ± 0.21	7.40 ± 0.92	0.04 ± 0.01	4.27 ± 2.05	3.59 ± 1.87	3.47 ± 1.85	0.12 ± 0.03	7.01 ± 1.89
	SY	31.00 ± 1.24	30.93 ± 3.48	6.8 ± 0.95	9.21 ± 0.31	9.78 ± 1.71	0.05 ± 0.01	4.91 ± 0.98	6.26 ± 1.69	3.02 ± 1.47	0.12 ± 0.02	79.62 ± 13.64
Autumn	SZ	25.64 ± 3.06	13.98 ± 2.96	4.00 ± 1.49	7.17 ± 0.44	8.11 ± 0.87	0.05 ± 0.03	2.24 ± 0.79	2.61 ± 0.83	2.62 ± 1.63	0.09 ± 0.03	7.68 ± 3.36
	SY	26.54 ± 4.17	23.63 ± 4.11	4.86 ± 1.69	7.78 ± 0.78	8.10 ± 1.15	0.04 ± 0.02	2.56 ± 0.72	4.51 ± 1.07	2.69 ± 1.55	0.08 ± 0.03	37.41 ± 16.02
Winter	SZ	18.59 ± 2.23	11.30 ± 3.43	3.00 ± 0.46	7.79 ± 0.69	10.38 ± 0.4	0.05 ± 0.05	1.93 ± 0.48	3.14 ± 1.36	4.74 ± 1.93	0.08 ± 0.03	16.93 ± 5.56
	SY	18.83 ± 2.12	20.23 ± 2.45	4.9 ± 1.61	7.84 ± 0.53	9.43 ± 1.21	0.06 ± 0.08	2.43 ± 0.17	4.55 ± 1.55	4.06 ± 1.96	0.1 ± 0.03	37.59 ± 9.04

All values are given as mean values with standard error

Water temperature is an important factor to algal growth, and temperature of the two reservoirs is similar as above 18 °C all the year, and obviously higher than the water temperature in the northern China (about 10–15 °C) [41, 42], which is more conducive to the growth of phytoplankton. However, by comparing SZ and SY, we found that there is a difference between SZ and SY in 2-MIB and algae. The average concentration of 2-MIB in SY is 48.06 ng/L and the peak value even more than 60 ng/L, while the 2-MIB of SZ is always below its odor threshold (10 ng/L) (Additional file 1: Figure S6). In terms of algae, the algae counts in SY are also significantly higher than those in SZ, especially for *Pseudanabaena* sp. This means that the environmental conditions of SY are more suitable for the algae growth.

**Fig. 6:**
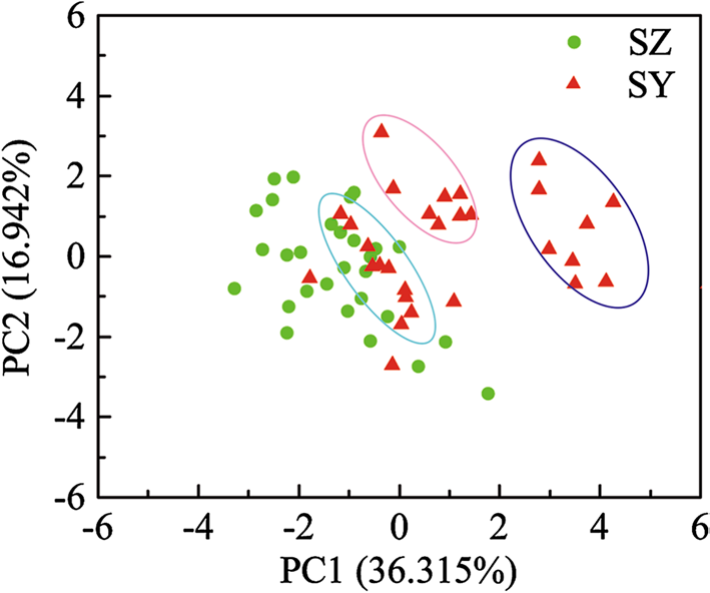
PCA plot based on the physio-chemical variables in SZ and SY. The purple ellipse is mainly for July to October data, the pink ellipse is mainly for November to February data, and the blue ellipse is mainly for March to June data

Principal compound analysis (PCA) was used to analyze the difference of physio-chemical variables in SZ and SY to find the key factors cause of the difference in phytoplankton (Fig. 6). The first two PCs explained 53.257% of the variance together; the parameters of water temperature, pH, DO, total phosphorus (TP) and total nitrogen (TN) primarily contribute to PC1, while chroma, turbidity, and COD_Mn_ primarily contribute to PC2.

As shown in Fig. 6, the parameters data of SY can be well separated in the direction of PC1, and the data derived from different season were clustered together. It indicates that the water quality of SY changes greatly with seasons. In contrast, the water parameters data of SZ cannot be well separated in the PC1 or PC2 direction, which indicates that the water quality of SZ is stable throughout the year and little fluctuation occurs therein. It is worth mentioning that SY and SZ are at similar water temperature, TP values and TN values; this shows that these factors are not limiting factors for algae growth in the SY. The two reservoirs are different in turbidity, DOC, COD_Mn_, pH, chroma and DO which may be important factors for algae growth. However, previous studies showed that the change of pH, chroma and DO is mainly caused by phytoplankton growth, so the turbidity, DOC and COD_Mn_ may be the main reasons leading to the difference of the algae in the two reservoirs.

The environment around SZ and SY is quite different. SZ is located in the original Shenzhen Special Economic Zone (SEZ) and surrounded by East Lake park with a cleaner environment. While SY is located outside the original SEZ, there are many residential areas and industrial areas around it, and the drainage pipe network is not well designed and planned, so some sewage enters into the reservoir. This may lead to a higher COD_Mn_ and DOC in SY. On the other hand, the raw water of Shenzhen City comes mainly from the Dongjiang River. The raw water enters SZ directly, while the raw water entering the SY must flow through the Xili reservoir and the Tiegang reservoir. The water quality may be thus affected. Therefore, strengthening the protection of SY and reducing external pollution may be one of the important means able to reduce the growth of the *Pseudanabaena* sp. in the reservoir.

**Fig. 7:**
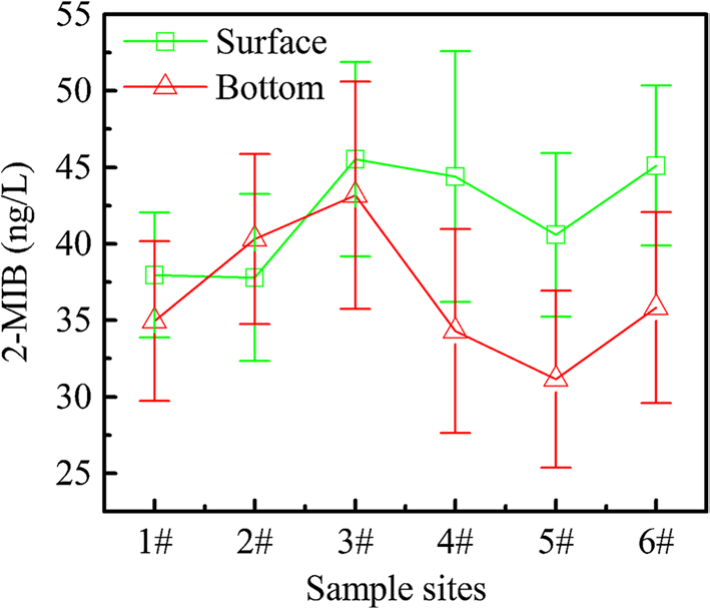
2-MIB distribution in surface and bottom of different sites in SY

**Fig. 8:**
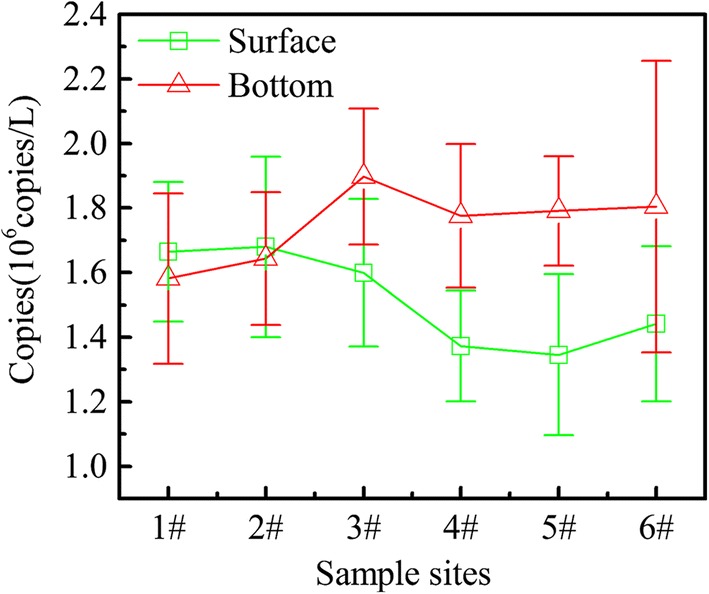
*mic* distribution in surface and bottom of different sites in SY

As the concentration of 2-MIB is always at high level in SY from April to July each year, several previous odor events also occurred in this period. So additional sampling sites (1–6#) were added during this period and the results of 2-MIB and gene *mic* distribution in these sites are shown in Figs. 7 and 8.

From Fig. 8, the concentration of 2-MIB varied greatly across different sites. In the surface layer of the reservoir, the concentration of 2-MIB at 4–6# which away from the water inlet is much higher than 1–3# close to the inlet, while the 2-MIB distribution in the bottom layer presents the opposite phenomenon. In the vertical direction, the 2-MIB concentration in surface and bottom is at same level in 1–3#, while the 2-MIB concentration far from the inlet in the surface is higher than that on the bottom (45.5 ± 2.3 ng/L and 37.4 ± 3.6 ng/L, respectively). The distribution of *mic* genes is significantly different from 2-MIB, showing the opposite situation in 3–6# (Fig. 8). At the 1–2# sampling sites, the number of *mic* in bottom layer is basically same as that in the surface layer, which is very similar to the distribution of 2-MIB. However, at 3–6#, the number of *mic* for the samples taken at the bottom layer was much higher if compared to the corresponding surface layer water samples. This may be attributed to the fact that the *Pseudanabaena* sp. is one typical benthic alga, which prefers to grow near the sediment to obtain enough nutrients [43]. The water parameters pertinent to the surface, and bottom, of SY are listed in Additional file 1: Table S2.

As the 1–2# sampling sites are close to water inlet, these areas are shallow (about 3 m depth) and water flow is fast; the water was well mixed. This may be the main reason for the similar concentration of 2-MIB and number of *mic* in the surface and on the bottom. While the water depth is about 10 m, even greater than 15 m at the 3–6# sampling sites, the water qualities at these sites vary with depth which may lead to a different distribution of *mic* at different depths in the reservoir. It should be noted that the *mic* gene can only represent the potential for 2-MIB production, while the mechanism of 2-MIB synthesis of internal processes remains vague; the regulation of gene expression in RNA level may be influenced by many factors, such as light and water temperature [38]. Therefore, higher number of *mic* does not necessarily correlate with a higher concentration of 2-MIB. Zhang et al. [38] have suggested that increasing temperature could increase the proportion of dissolved 2-MIB in the water and the water temperature on the surface layer is higher than that at the bottom; this may promote the release of 2-MIB from the surface layer *Pseudanabaena* sp. During this period, continuous rainfall often occurs and the concentration of 2-MIB often increased significantly on the sunny day after rain. GPP is the precursor of 2-MIB, also being the intermediate precursor of chlorophyll-a, and light plays a key role in regulating this competition [44, 45]. When cyanobacterial cells were at light stressed conditions, the use of GPP shifted to 2-MIB synthesis. The average proportion of extracellular 2-MIB to total 2-MIB gradually increased up to 68.42% when increasing the light intensity from 10 to 85 μmol photons m^−2^ s^−1^ [38]. Noticeably, more water would enter the reservoir during rainy season, increasing the mix of water, which might bring the *Pseudanabaena* sp. to the surface from the deep area. The *Pseudanabaena* sp. is directly exposed to strong, damaging irradiances during the sunny days, the ensuing algal cell death may cause cell rupture and decay led to intracellular 2-MIB release.

Underwater light availability is one of the most important growth factors for cyanobacteria and reduces significantly with the water level increase (about 4.2–16% of light intensity will be reduced for each 10 cm water level increase in China) [18]. Some scholars proposed reducing the light intensity by changing the depth of the reservoir can control the growth of algae [42]. However, the increase of water level may also cause nutrient dilution and decrease of water temperature, which is not only inhibit the growth of algae, but also detrimental to the production and release of 2-MIB in the cell [46–48]. Therefore, the principle and actual regulation effect of this method should be studied and verified in the future.

It is worth noting that providing effective early warning for water plants is very important. There are many models to predict odor in other areas [49–51]; however, given the different environments in different regions, one warning model may not be applicable in all places. In our future work, efforts should be made to enhance the water quality monitoring of the reservoir, so as to establish accurate early warning models for better incident prediction, warning, and prevention.

## Conclusions

This is the first systematic study on the T&O problems in drinking water in Shenzhen City. An integrated approach including chemical analysis (GC × GC–TOFMS), sensory evaluation (FPA) and molecular biological method (qPCR) was employed. It is confirmed that 2-MIB is the odor-caused substance in Shiyan reservoir and *Pseudanabaena* sp. is the main 2-MIB producer. Compared with nitrogen and phosphorus, COD_Mn_ and DOC had higher effects on *Pseudanabaena* sp. growth in Shiyan Reservoir, thus strengthening environmental protection around the reservoir to reduce the COD_Mn_ and DOC input which might be a useful measure for controlling the growth of *Pseudanabaena* sp. We also demonstrated that *Pseudanabaena* sp. is a benthic filamentous algae; the algae growth and 2-MIB release are affected by temperature and light. The risk of sudden increase of 2-MIB will be reduced by raising the depth of water in the reservoir. Our study will improve the understanding of T&O problems in Shenzhen City, as well as in other hot and humid areas in China.

## Additional file

**Additional file 1: Figure S1.** The change of total phytoplankton cell number in SZ and SY during the monitoring period. **Figure S2.** Relative abundance of phytoplankton in two reservoirs during the monitoring period. (a): Shenzhen Reservoir, (b): Shiyan Reservoir. **Figure S3.** Microscope picture of main algae in two reservoirs. (a): *Pseudanabaena* sp., (b): *Cylindrospermopsis raciborskii*, (c): *Synedra*, (d): *Melosira granulata* (*Ehr.*) *Ralfs*, (e): *Cyclotella* sp., (f): *Cryptomonas*, (g): *Naviculaceae*, (h): *Rhizosolenia*, (i): *Nitzschia*. **Figure S4.** Inter-annual variability of 2-MIB and chlorophyll-a in SY. The red triangle and the green circle represent 2-MIB and chlorophyll-a respectively, when the *Pseudanabaena* sp. is the dominant species. **Figure S5.** Agarose gel electrophoresis result of genomic DNA and PCR products before and after purification operation. (a): genomic DNA extract from FACHB1277, (b): The PCR product primed by MIB-R(f/r) before purification operation, (c): The PCR product primed by MIB-R(f/r) after purification operation. **Figure S6.** The concentration of 2-MIB changing in SZ and SY during the monitoring period. **Table S1.** The different odor type solution in re-test. **Table S2.** The water parameters of surface and bottom in different sampling sites in SY.

## Abbreviations

T&O: taste and odor
2-MIB: 2-methylisoborneol
GC × GC–TOFMS: comprehensive two-dimensional gas chromatography with time-of-flight mass spectrometry
FPA: flavor profile analysis
qPCR: quantitative real-time polymerase chain reaction
GC/MS: gas chromatography and mass spectrometry
OTC: odor threshold concentration
OAV: odor activity value
PCA: principal compound analysis
COD_Mn_: oxygen consumption
DOC: dissolved organic carbon
