# Supplementary material for: Source water odor in one reservoir in hot and humid areas of southern China: occurrence, diagnosis and possible mitigation measures
Source: Environ Sci Eur. 2018 Nov 27;30(1):45. doi: 10.1186/s12302-018-0175-8 (PMC6267717; doi:10.1186/s12302-018-0175-8)
Supplement: Supplementary file 1 — Additional file 1: Figure S1. The change of total phytoplankton cell number in SZ and SY during the monitoring period. Figure S2. Relative abundance of phytoplankton in two reservoirs during the monitoring period. (a): Shenzhen Reservoir, (b): Shiyan Reservoir. Figure S3. Microscope picture of main algae in two reservoirs. (a): Pseudanabaena sp., (b): Cylindrospermopsis raciborskii, (c): Synedra, (d): Melosira granulata (Ehr.) Ralfs, (e): Cyclotella sp., (f): Cryptomonas, (g): Naviculaceae, (h): Rhizosolenia, (i): Nitzschia. Figure S4. Inter-annual variability of 2-MIB and chlorophyll-a in SY. The red triangle and the green circle represent 2-MIB and chlorophyll-a respectively, when the Pseudanabaena sp. is the dominant species. Figure S5. Agarose gel electrophoresis result of genomic DNA and PCR products before and after purification operation. (a): genomic DNA extract from FACHB1277, (b): The PCR product primed by MIB-R(f/r) before purification operation, (c): The PCR product primed by MIB-R(f/r) after purification operation. Figure S6. The concentration of 2-MIB changing in SZ and SY during the monitoring period. Table S1. The different odor type solution in re-test. Table S2. The water parameters of surface and bottom in different sampling sites in SY. [file 12302_2018_175_MOESM1_ESM.docx]

**Additional file**





Figure S1. The change of total phytoplankton cell number in SZ and SY during the monitoring period.







(a) (b)

Figure S2. Relative abundance of phytoplankton in two reservoirs during the monitoring period. (a): Shenzhen Reservoir, (b): Shiyan Reservoir.


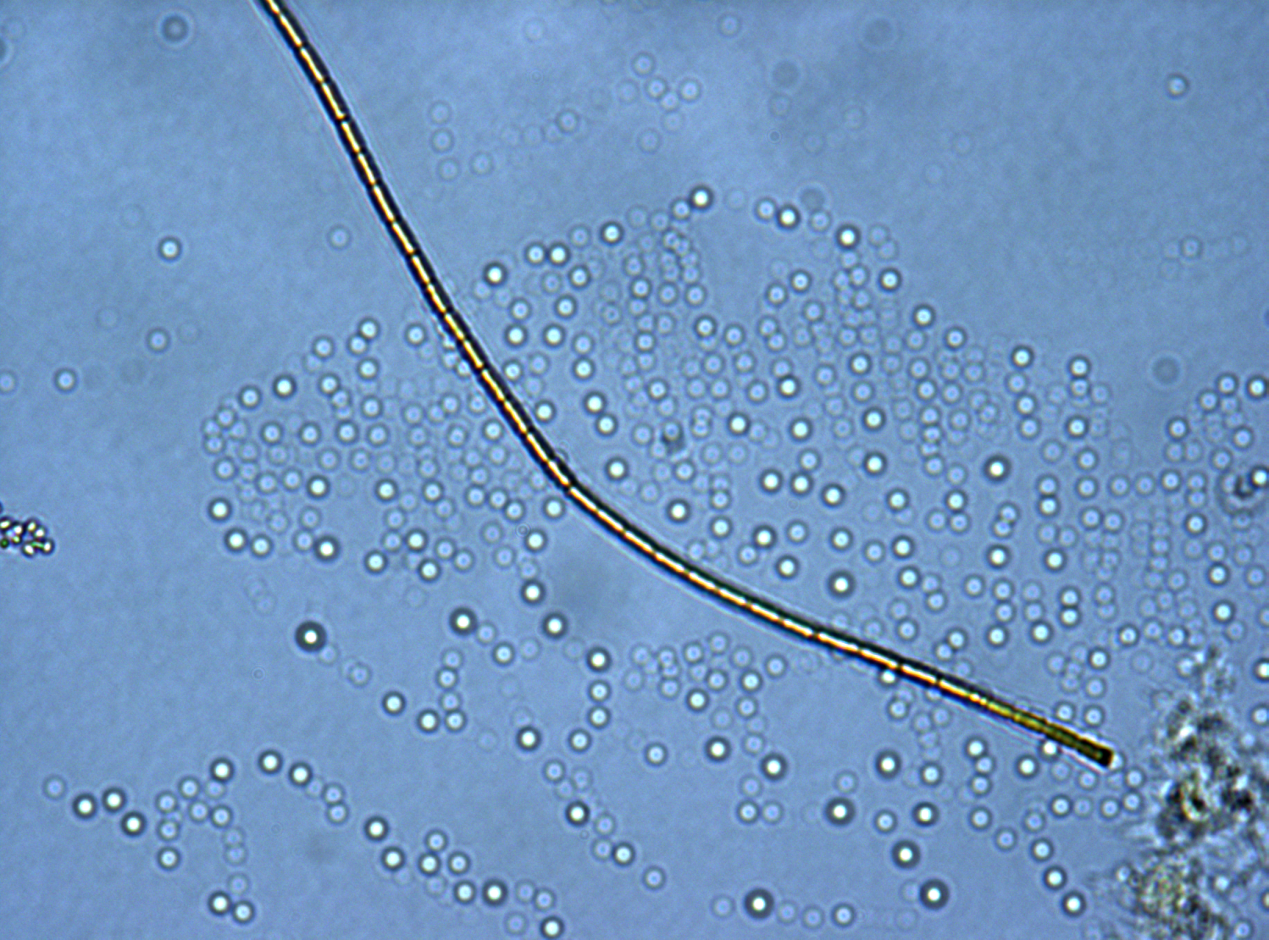

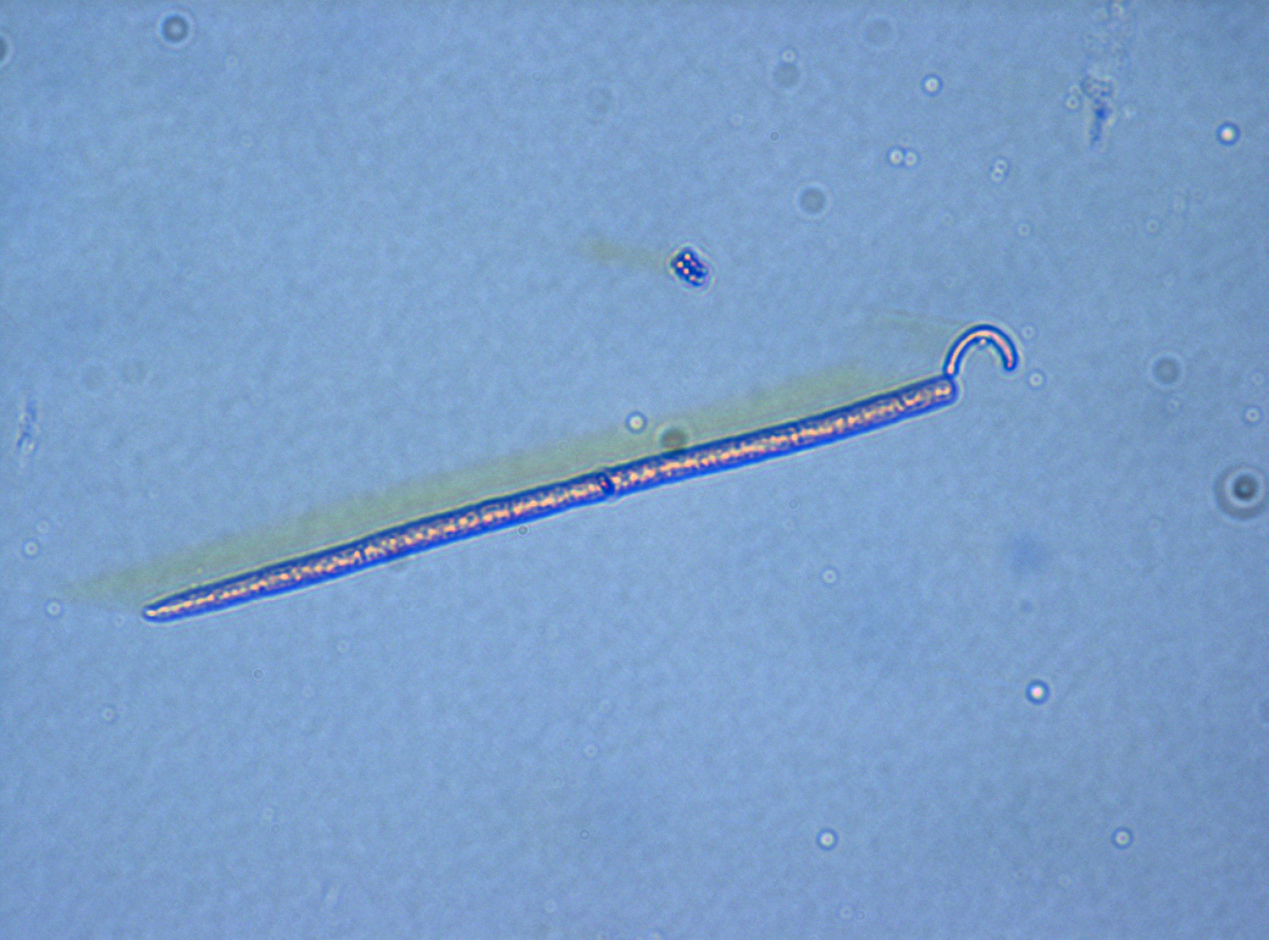

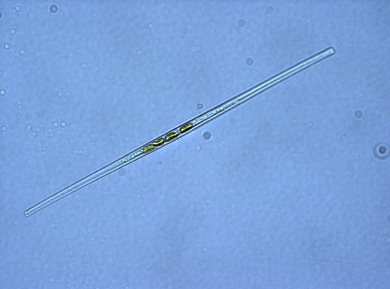
(a) (b) (c)


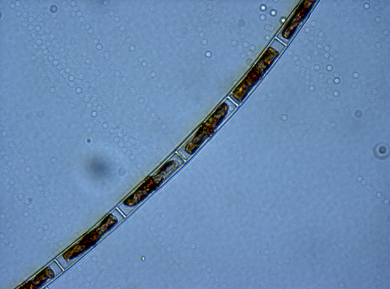

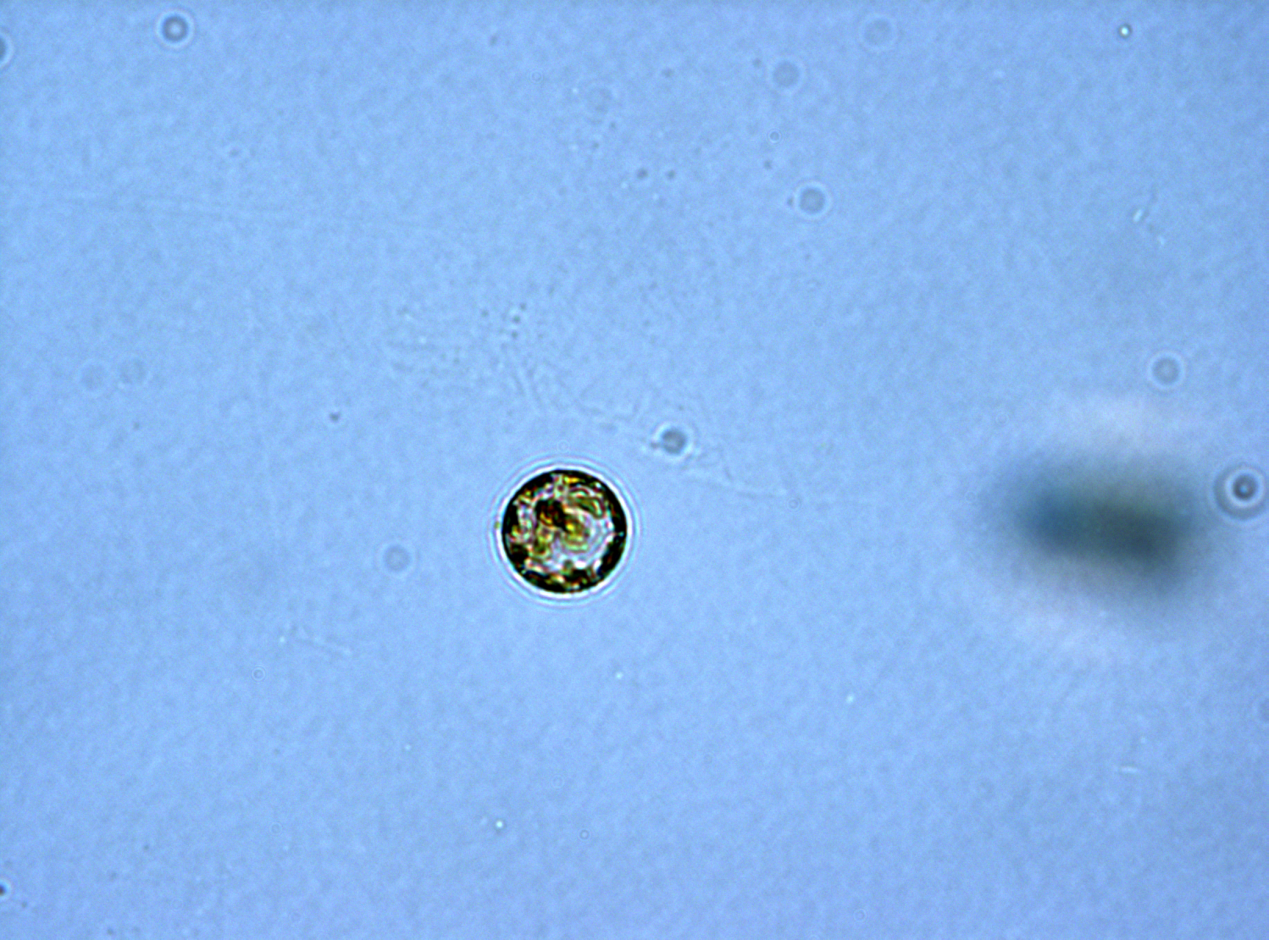

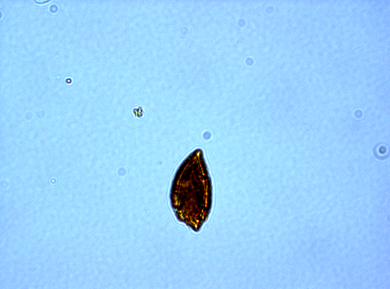


(d) (e) (f)


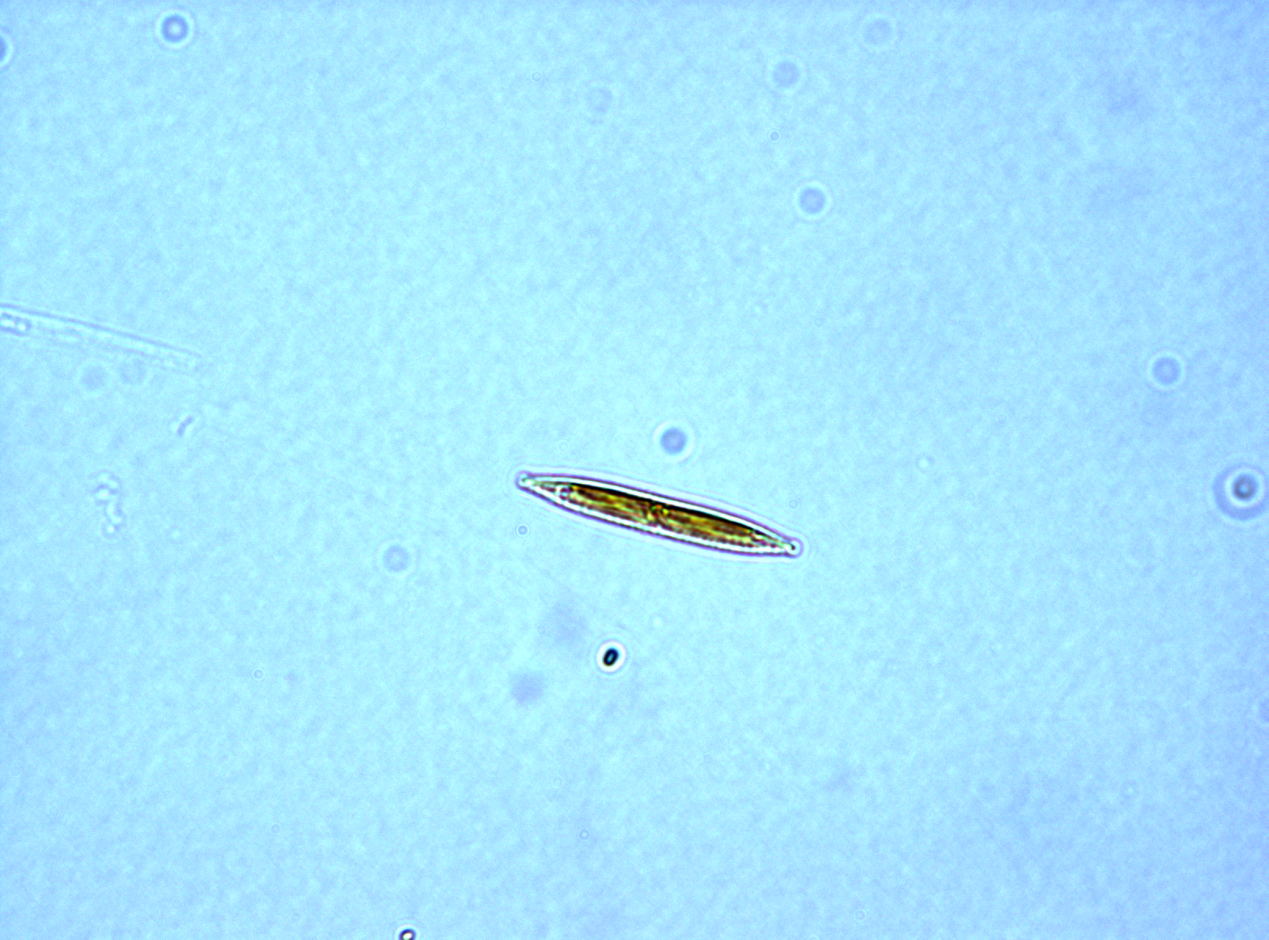

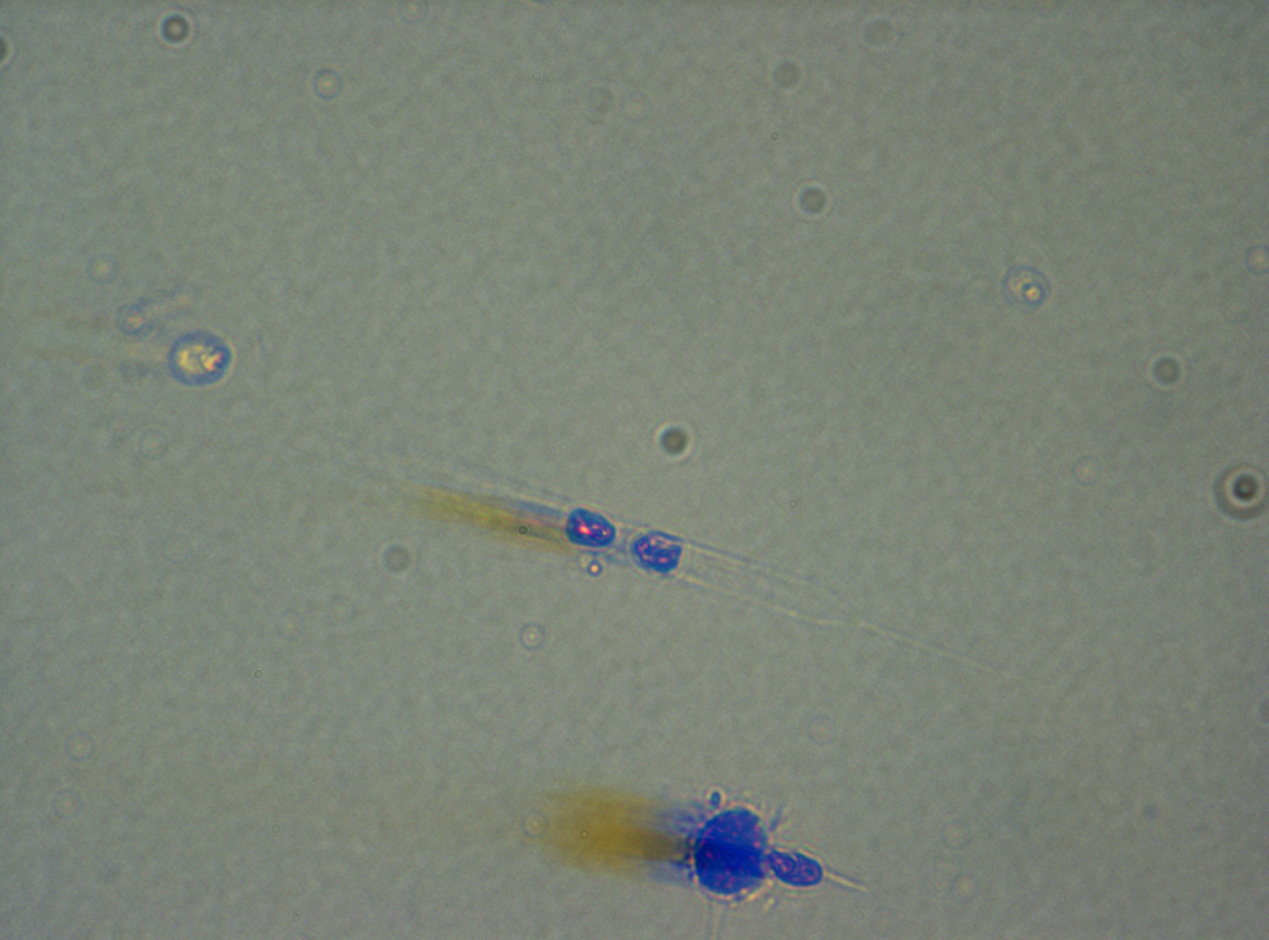

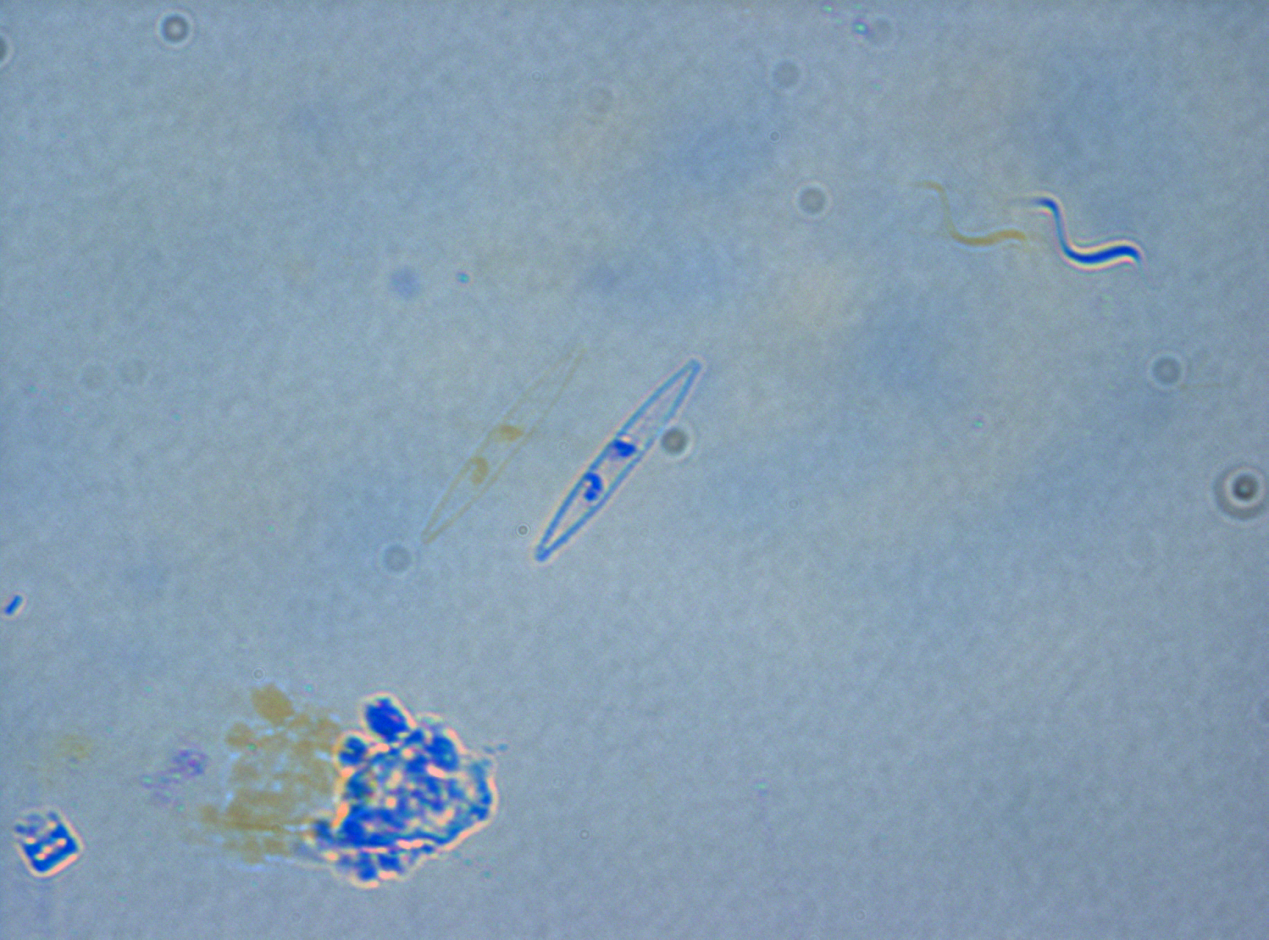
(g) (h) (i)

Figure S3. Microscope picture of main algae in two reservoirs. (a): *Pseudananbaena* sp.,(b): *Cylindrospermopsis raciborskii*, (c): *Synedra*, (d): *Melosira granulata(Ehr.) Ralfs*, (e): *Cyclotella* sp., (f): *Cryptomonas*, (g): *Naviculaceae*, (h): *Rhizosolenia*, (i): *Nitzschia*.





Figure S4. Inter-annual variability of 2-MIB and Chlorophyll-a in SY. The red triangle and the green circle represent 2-MIB and chlorophyll-a respectively, when the *Pseudananbaena* sp*.* is the dominant species.


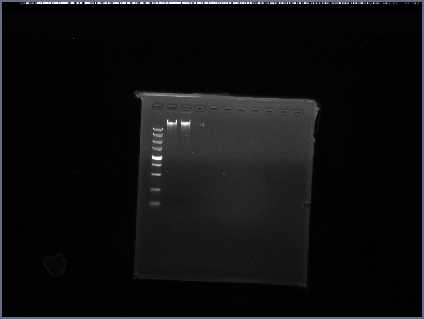

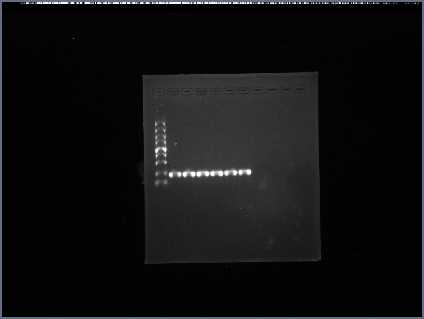

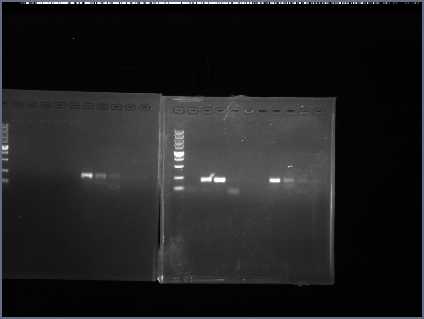


(a) (b) (c)

Figure S5. Agarose gel electrophoresis result of cDNA and PCR products before and after purification operation. (a): genomic DNA extract from FACHB1277, (b): The PCR product primed by MIB-R(f/r) before purification operation, (c): The PCR product primed by MIB-R(f/r) after purification operation.


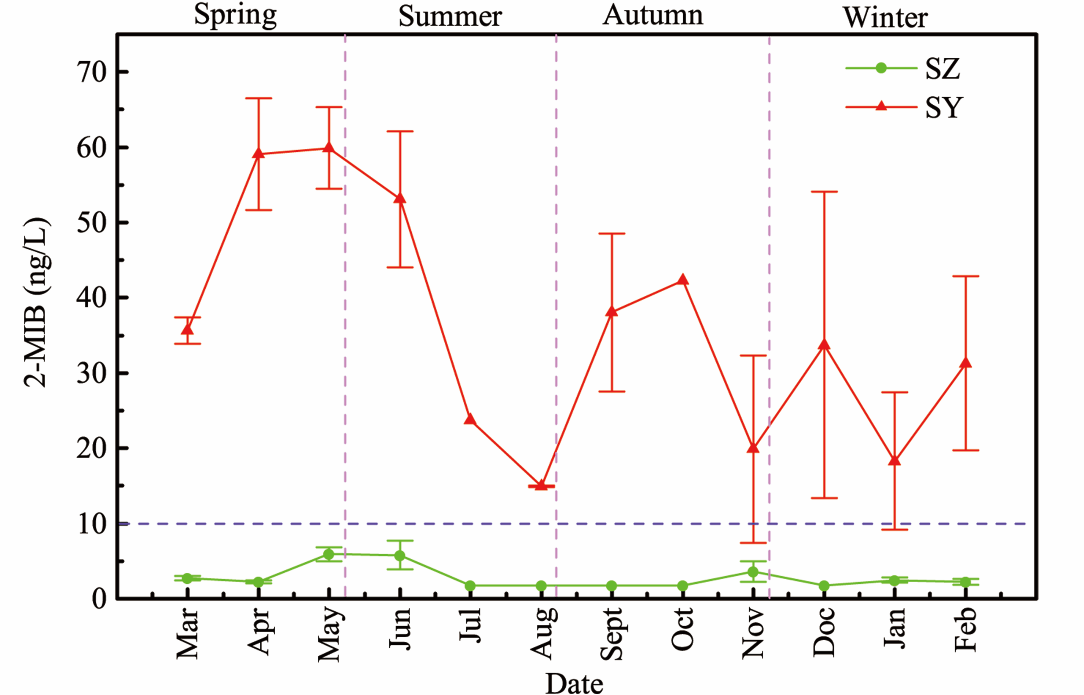


Figure S6. The concentration of 2-MIB changing in SZ and SY during the monitoring period

Table S1 The different odor type solution in re-test

| Compounds | Odor type | Concentration(ng/L) |
| --- | --- | --- |
| 2-MIB&Geosmin | Earthy-musty | 50 |
| 2-Methyl-phenol | Medicinal odor | 50 |
| NaClO | Chlorine | 50000 |

Table S2 The water parameters of surface and bottom in different sampling sites in SY

|  | 1# | | 2# | | 3# | | 4# | | 5# | | 6# | |
| --- | --- | --- | --- | --- | --- | --- | --- | --- | --- | --- | --- | --- |
|  | Surface | Bottom | Surface | Bottom | Surface | Bottom | Surface | Bottom | Surface | Bottom | Surface | Bottom |
| Water Temp.  (^o^C) | 28.5±1.18 | 27.3±0.72 | 28.53±1.18 | 27.4±0.87 | 28.23±1.2 | 26.13±0.63 | 28.67±1.25 | 24.2±0.67 | 28.6±1.27 | 23.5±0.73 | 27.8±1.32 | 26.1±0.64 |
| Chroma | 22.38±1.25 | 27.84±3.09 | 24.95±2.06 | 24.38±1.58 | 21.76±1.04 | 19.54±0.87 | 20.18±0.5 | 19.71±0.57 | 20.33±0.96 | 25.42±2.43 | 18.51±1.37 | 18.74±1.87 |
| Turbidity  (NTU) | 6.86±0.58 | 7.05±1.07 | 6.59±0.63 | 7.21±0.82 | 5.09±0.2 | 5.25±0.26 | 5.08±0.24 | 5.05±0.21 | 4.15±0.24 | 5.84±0.41 | 4.05±0.21 | 4.56±0.38 |
| pH | 8.12±0.11 | 8.33±0.14 | 8.49±0.16 | 8.19±0.18 | 8.62±0.1 | 7.58±0.09 | 8.53±0.13 | 7.55±0.16 | 8.51±0.15 | 7.33±0.11 | 8.53±0.22 | 7.92±0.25 |
| DO  (mg L^-1^) | 8.42±0.22 | 8.32±0.19 | 8.75±0.34 | 8.46±0.2 | 8.5±0.27 | 7.5±0.14 | 8.27±0.3 | 6.99±0.3 | 8.55±0.2 | 6.76±0.36 | 8.39±0.22 | 7.77±0.04 |
| UV_254_ | 0.0401±0.002 | 0.0414±0.0024 | 0.0383±0.0009 | 0.0366±0.0017 | 0.0362±0.0007 | 0.0357±0.0005 | 0.0352±0.0008 | 0.0357±0.0012 | 0.0364±0.0009 | 0.0513±0.0063 | 0.0386±0.0023 | 0.0386±0.0023 |
| DOC  (mg L^-1^) | 2.75±0.04 | 2.74±0.11 | 2.61±0.04 | 2.65±0.09 | 2.57±0.04 | 2.49±0.07 | 2.84±0.1 | 2.88±0.2 | 2.99±0.21 | 2.83±0.22 | 2.78±0.1 | 2.57±0.12 |
| COD_Mn_  (mg L^-1^) | 2.75±0.04 | 2.74±0.11 | 2.61±0.04 | 2.65±0.09 | 2.57±0.04 | 2.49±0.07 | 2.84±0.1 | 2.88±0.2 | 2.99±0.21 | 2.83±0.22 | 2.78±0.1 | 2.57±0.12 |
| TN  (mg L^-1^) | 4.22±0.09 | 4.28±0.08 | 4.31±0.08 | 4.33±0.05 | 4.29±0.09 | 4.36±0.1 | 4.29±0.12 | 4.45±0.1 | 4.28±0.08 | 4.46±0.15 | 4.35±0.14 | 4.32±0.14 |
| TP  (mg L^-1^) | 132.55±16.88 | 148.6±17.79 | 110.65±15.81 | 109.45±25.68 | 103.34±13.14 | 91.75±6.35 | 93.35±8.56 | 110.36±9.96 | 84.47±13.04 | 109.42±10.59 | 91.29±11.05 | 79.38±12.6 |
| Chl-a  (μg L^-1^) | 36.67±5.56 | 32.14±2.81 | 47.03±7.28 | 32.16±1.02 | 41.55±5.18 | 25.57±0.47 | 33.89±3.9 | 27.7±2.81 | 31.24±2.67 | 25.36±2 | 25.42±3.63 | 24.83±4.68 |
